# Microbiome response in an urban river system is dominated by seasonality over wastewater treatment upgrades

**DOI:** 10.1186/s40793-023-00470-4

**Published:** 2023-02-19

**Authors:** Sho M. Kodera, Anukriti Sharma, Cameron Martino, Melissa Dsouza, Mark Grippo, Holly L. Lutz, Rob Knight, Jack A. Gilbert, Cristina Negri, Sarah M. Allard

**Affiliations:** 1 Scripps Institution of Oceanography, University of California San Diego, La Jolla, CA USA; 2 Department of Quantitative Health Sciences, Lerner Research Institute, Cleveland Clinic, Cleveland, OH USA; 3 Department of Pediatrics, University of California San Diego, La Jolla, CA USA; 4 Bioinformatics and Systems Biology Program, University of California San Diego, La Jolla, CA USA; 5 Merck Inc., Cambridge, MA USA; 6 Environmental Science Division, Argonne National Laboratory, University of Chicago, Lemont, IL USA; 7 Department of Immunology and Microbiology, The Scripps Research Institute, La Jolla, CA USA; 8 Center for Microbiome Innovation, Jacobs School of Engineering, University of California San Diego, La Jolla, CA USA; 9 Department of Computer Science and Engineering, Jacobs School of Engineering, University of California San Diego, La Jolla, CA USA; 10 Department of Bioengineering, University of California San Diego, La Jolla, CA USA

**Keywords:** Wastewater, Microbiome, Dynamics, 16S rRNA gene sequencing, Fecal coliform

## Abstract

**Background:**

Microorganisms such as coliform-forming bacteria are commonly used to assess freshwater quality for drinking and recreational use. However, such organisms do not exist in isolation; they exist within the context of dynamic, interactive microbial communities which vary through space and time. Elucidating spatiotemporal microbial dynamics is imperative for discriminating robust community changes from ephemeral ecological trends, and for improving our overall understanding of the relationship between microbial communities and ecosystem health. We conducted a seven-year (2013–2019) microbial time-series investigation in the Chicago Area Waterways (CAWS): an urban river system which, in 2016, experienced substantial upgrades to disinfection processes at two wastewater reclamation plants (WRPs) that discharge into the CAWS and improved stormwater capture, to improve river water quality and reduce flooding. Using culture-independent and culture-dependent approaches, we compared CAWS microbial ecology before and after the intervention.

**Results:**

Examinations of time-resolved beta distances between WRP-adjacent sites showed that community similarity measures were often consistent with the spatial orientation of site locations to one another and to the WRP outfalls. Fecal coliform results suggested that upgrades reduced coliform-associated bacteria in the effluent and the downstream river community. However, examinations of whole community changes through time suggest that the upgrades did little to affect overall riverine community dynamics, which instead were overwhelmingly driven by yearly patterns consistent with seasonality.

**Conclusions:**

This study presents a systematic effort to combine 16S rRNA gene amplicon sequencing with traditional culture-based methods to evaluate the influence of treatment innovations and systems upgrades on the microbiome of the Chicago Area Waterway System, representing the longest and most comprehensive characterization of the microbiome of an urban waterway yet attempted. We found that the systems upgrades were successful in improving specific water quality measures immediately downstream of wastewater outflows. Additionally, we found that the implementation of the water quality improvement measures to the river system did not disrupt the overall dynamics of the downstream microbial community, which remained heavily influenced by seasonal trends. Such results emphasize the dynamic nature of microbiomes in open environmental systems such as the CAWS, but also suggest that the seasonal oscillations remain consistent even when perturbed.

**Supplementary Information:**

The online version contains supplementary material available at 10.1186/s40793-023-00470-4.

## Introduction

High quality fresh water is a critical natural asset that is under increasing risk of overuse and contamination by anthropogenic influences [57]. With over half of the world’s total population living in urban areas, urban waterways are particularly influenced by human activity and act as a liaison between humans and the natural environment [15]. Historically, cultured microorganisms have been utilized as metrics of water quality due to their traceability to potential sources of contamination. Concentrations of *Escherichia coli* or, more broadly, coliform-forming bacteria, have typically been used as indicators for fecal contamination in recreational, agricultural, and drinking water [34]. Although valuable, such approaches can vary in both reliability and accurate representation of human pathogen levels [6, 8, 33, 40]. Furthermore, culture-based methods do not capture changes in the ecological structure of the community as a whole, which can provide valuable insights into ecosystem health [10, 46, 47]. Bacterial pathogens and fecal indicator taxa do not exist in isolation, rather, they exist within expansive, interactive communities of diverse and abundant microbial members [12, 24]. Therefore, improving our understanding of the relationship between microbiology and water quality may require a more holistic examination of the microbial community.

Microbial communities in aquatic systems are known to be highly responsive to environmental variables that vary across space and time (e.g. temperature, nutrient availability, hydrology, metal contamination, and contrasting land-use; [49, 50, 53, 56, 59, 60]. Therefore, management efforts of water resources must take into account such spatio-temporal dependencies, particularly in fluctuating environments such as riverine systems. Moreover, riverine systems are recipients of diverse microbial inputs that influence their ecology. As an example, the Chicago Area Waterway System (CAWS) is an extensive, highly engineered urban river system that consists of over 76 miles of man-made canals and modified natural streams (Additional file 1: Fig. S1). Like many urban waterways, the CAWS receives treated wastewater from multiple reclamation plants. In 2016, advanced disinfection systems at two of these wastewater reclamation plants (WRPs) were implemented to significantly improve the treatment of wastewater before discharge into the CAWS. A seven-channel UV chamber system was installed at O’Brien WRP, while a chlorination-dechlorination system was installed at Calumet WRP. Simultaneously, the segment of the Tunnel And Reservoir Plan (TARP) associated with the Calumet WRP was implemented. The TARP is a tunnel system that captures combined untreated sewage and stormwater from the surrounding area, preventing Combined Sewer Overflow (CSO) directly discharges into the CAWS during rainy weather and high flow conditions.

In collaboration with Chicago’s Metropolitan Water Reclamation District (MWRD) we conducted a 7-year investigation (2013–2019) of the CAWS microbiome, collecting monthly samples of water and sediment across 12 sites as well as untreated sewage and treated effluent samples from two wastewater reclamation plants (WRPs). We analyzed these samples with 16S rRNA amplicon sequencing and fecal coliform enumeration, and continually monitored relevant physicochemical characteristics at each site. Notably, the sampling period of our study coincided with the implementation of the two major water quality improvement efforts at the CAWS. The implementation of these initiatives during our longitudinal study provided a unique opportunity to examine how microbial community dynamics in a wastewater-impacted water system may be affected when presented with substantial wastewater management upgrades.

The primary goals of this study were to a) characterize the microbial communities of the CAWS and its spatio-temporal dynamics across 12 riverine sites and 2 wastewater treatment plants for 63 timepoints over 7 years, b) compare such characterizations to the trends found in a traditional, culture-dependent approach to measuring water quality, and c) examine the impact of water quality improvement interventions on the microbial ecology of the CAWS. The combination of sequencing data, fecal coliform data, and physicochemical data thus enabled us to parameterize the microbial ecology of the CAWS to gain nuanced insights into how environmental disturbance impacts the ecology of this already dynamic microbial system.

## Materials and methods

Overall, water and sediment samples were collected from 12 sites along the CAWS, while raw sewage influent and treated effluent samples were collected from the O’Brien and Calumet WRPs (Additional file 1: Fig. S1, Table S1). Water and effluent samples were collected monthly from May 2013—September 2019, sewage samples were collected monthly from June 2014—September 2019, and sediment samples were collected from May 2013—November 2018. Samples were not collected during the months of December, January, and February due to weather conditions as well as due to sampling not being covered by the WRPs’ National Pollution Discharge Elimination System (NPDES) Permit during these months. We collected a total of 2,306 samples: 260 effluent samples, 558 sediment samples, 928 water column samples, 88 sewage samples, and 472 technical controls (bottle, filter, equipment blanks). We utilized 16S rRNA gene amplicon sequencing on these samples along with fecal coliform counts and in-situ physicochemical measurements to characterize the microbial ecology of the CAWS from 2013 to 2019.

### Sample collection

All CAWS locations were sampled by MWRD personnel for water and sediment. Sewage and effluent samples were also collected by MWRD personnel at the WRPs. River water samples used for microbiome analysis were collected at the surface, while river water samples used for fecal coliform counts were collected at a depth of three feet due to sampling device differences. Sediment samples were collected using either a ponar sampling device or a hand scoop. Raw sewage grab samples were collected after the fine-screening stage, but before primary settling. Final effluent grab samples were collected after the final stage of treatment, before discharge into the receiving water body. For each sampling effort, either 500 mL of liquid (water, sewage, and effluent) or 100 g of sediment were collected in sterile containers for microbiome analysis.

Temperature, pH, conductivity, and turbidity of water samples were measured using a handheld YSI multiparameter digital water quality meter. NO_2_^−^/NO_3_^−^ ratios, NH_3_, and PO_4_^3−^ values were measured using a Lachat Quickchem 8500 Series 2.0 instrument, using EPA 353.2 Rev 2.0, EPA 350.1 Rev 2.0, and EPA 365.4 reference methods, respectively. Chlorophyll measurement protocols were adapted from standard methods used in the Examination of Water and Wastewater method 10,200 H, with measurements between 2013 and 207 being made using a Beckman DU-640 spectrophotometer and measurements in 2017–2019 being made using a Thermoscientific Genesys 10 s UV–VIS spectrophotometer. Dissolved oxygen was initially measured using the Winkler method between 2013 and 2018, while after 2018 the measurement protocol was transitioned to using a HACH HQd portable meter with a luminescent DO probe. Sediment samples were stored in polypropylene containers at 4 °C. Water samples (200 mL for CAWS and effluent samples and 25 mL for raw sewage samples) were filtered in duplicate using 0.22 Micron Mixed Cellulose Ester filters, and filters were aseptically transferred to labeled sterile 50 mL tubes and stored at – 80 °C. All water and sediment sample aliquots were removed from – 80 °C, transferred to the lab on ice, and then stored at – 80 °C until thawing for processing for sequencing. Samples aliquoted for fecal coliform analysis were analyzed on the same day as collection without freezing.

### Fecal coliform methods

Standard Methods 9222D Thermotolerant Fecal Coliform Membrane Filtration Procedure (18th edition) was used for fecal coliform testing of the water, sewage, and effluent samples. Volumes of samples used ranged from 10 mL (disinfected final effluent) to 0.001 mL (the lowest dilution of raw sewage). Petri dishes with mFC agar and filters were incubated for 24 ± 2 h at 44.5 ± 0.2 °C before counting colony forming units.

### 16S rRNA amplicon sequencing and bioinformatic processing

DNA was extracted using the protocol described by Marotz et al. [31] and the V4 region of the 16S rRNA gene was amplified using the protocol described by Caporaso et al. [7]. Briefly, we used region-specific primers (515F-806R) that included the Illumina flow cell adapter sequences and a 12-base barcode sequence for amplification of each 25 μl PCR reaction containing the following mixture: 12 μl of MoBio PCR Water (Certified DNA-Free,MoBio, Carlsbad, USA), 10 μl of 5-Prime HotMasterMix (1 ×), 1 μl of forward primer (5 μM concentration, 200 pM final), 1 μl of Golay Barcode Tagged Reverse Primer (5 μM concentration, 200 pM final), and 1 μl of template DNA. The conditions for PCR were as follows: 94 °C for 3 min to denature the DNA, with 35 cycles at 94 °C for 45 s, 50 °C for 60 s, and 72 °C for 90 s, with a final extension of 10 min at 72 °C to ensure complete amplification. Amplicons were quantified using PicoGreen (Invitrogen) assays on a plate reader, followed by clean up using the UltraClean® PCR Clean-Up Kit (MoBio, Carlsbad, USA) and quantification using Qubit readings (Invitrogen, Grand Island, USA). Amplicons were sequenced on an Illumina HiSeq2500 platform with paired-end sequencing at the Argonne National Laboratory Core Sequencing Facility according to protocols from the Earth Microbiome Project [54].

The raw sequence data was demultiplexed, trimmed, and processed using the open-source microbial study management platform Qiita [17]. Parameters for quality filtering included 75% consecutive high-quality base calls, a maximum of three low-quality consecutive base calls, zero ambiguous bases, and minimum Phred quality score of 3 as suggested previously [3]. Demultiplexed sequences were trimmed to 150 base pairs, and then selected for ASV (Amplicon Sequence Variant) picking using the Deblur pipeline v. 1.1.0 [1]. In the pipeline, de novo chimeras were identified and removed, artifacts (i.e. PhiX) were removed, and ASVs in less than 10 samples were removed for further analyses due to low representation. Blank samples were used as negative controls to determine the read count threshold for rarefaction. ASV tables were then rarefied to a sequencing depth of 1285 reads for downstream analyses (with the exception of differential abundance analyses described below), leading to the removal of 15 of 1834 non-blank samples which contained fewer reads than the set depth. Analysis was repeated at 2 higher rarefaction cutoffs, 3,000 and 10,000 reads per sample, confirming that the selected sampling depth produced comparable results while retaining more samples. Analysis was completed using both QIIME 2 2021.2 [4] and in R 3.4.2 via the phyloseq package [35].

### Alpha and beta diversity analysis

Alpha and beta diversity were calculated between different sample types as well as by year. Alpha diversity for all sample types was measured using Shannon’s index. Beta diversity was determined using both unweighted and weighted UniFrac distances [26, 27], which were ordinated using Principal Coordinate Analysis (PCoA). Statistical significance of the differences in microbial alpha diversity and beta diversity were assessed using paired t-tests with Benjamini–Hochberg corrections and permutational multivariate analysis of variance (PERMANOVA), respectively [2]. To examine the variability in community composition within sample types, beta dispersion values for each sample type were calculated using the mean value of unweighted UniFrac distances between all coordinates of individual samples to the coordinate of its respective sample type centroid. Statistical significance was assessed using paired t-tests with Benjamini–Hochberg corrections. Figures were generated using ggplot2() (https://github.com/tidyverse/ggplot2) in the R language (https://www.r-project.org/).

### Time series analyses

For each unique combination of sample type and site, the microbial community of a sample in early spring was designated as the “baseline” community for the sample type/site grouping (for effluent, water, and sediment, the baseline communities were set in March 2014; for sewage, the baseline communities were set in March 2015, as collection began after March 2014.). Next, the communities of all samples belonging to the same sample type/site grouping were compared to the baseline community through time using unweighted and weighted UniFrac distances. Distances to the baseline community (henceforth referred to as beta distances) were plotted through time to generate time series visualizations. Because no collections were conducted in the months of December, January, and February, missing time points were interpolated using linear spline interpolation [37]. Each time series was then decomposed into three components (trend, seasonality, and error) using STL (Seasonal-Trend decomposition procedure based on Loess,[9]. Next, analyses involving the identification of seasonal dynamics within sample types were then detrended according to the STL outputs (to remove any confounding effects of year-on-year changes in community dynamics). To test for the presence and strength of seasonality in each sample type, the detrended time series of a given sample type were combined across all sites and years, and were statistically tested for seasonality using Friedman’s tests (two-way analysis of variance by ranks) with Benjamini–Hochberg corrections. Analyses and visualizations were performed using the tsutils (https://github.com/trnnick/tsutils) and forecast [19] packages in R.

### Differential abundance analysis

To identify specific microbial ASVs driving the observed patterns of seasonality in water and effluent samples while accounting for the compositionality and uneven sampling depth inherent in 16S data, we conducted differential abundance analyses on the non-rarefied dataset using Songbird v. 1.0.4 through QIIME2 v. 2021.2 [38]. The differential abundance models were trained with the water and effluent samples using only the data from August and March time points, as we found in our time series analyses that the extremes of our seasonal patterns typically occurred at those two months for both water and effluent samples. Songbird differential rankings were visualized in Qurro [13] to identify the 10 most differentially abundant ASVs in March relative to August, and the 10 most differentially abundant ASVs in August relative to March, separately with both water and effluent samples. Once March-associated and August-associated ASVs were identified, we examined changes in their log-ratios across the entire dataset of water and effluent samples to test whether the identified ASVs follow a predictable seasonal gradient through time. We used the following formula:

$$ln (\frac{{\sum }_{n=1}^{10}March-associated ASV abundance}{{\sum }_{n=1}^{10}August-associated ASV abundance})$$

with a pseudo count of one applied to the table before taking the ratio to avoid undefined values.

### Time-resolved spatial analyses

To examine the impact of the WRP outflow and WRP upgrades on downstream microbial dynamics, we examined the time-resolved microbial dynamics of WRP-adjacent sites. Specifically, we examined spatial changes in microbiomes of water and sediment samples from sites immediately upstream, sites immediately downstream, and sites further downstream from both WRPs, as well as the WRP effluent samples. First, we used traditional ordination analyses with PCoA via weighted and unweighted UniFrac distances to visualize the targeted communities at pre- and post- intervention time points. We again used PERMANOVA to test for significant differences between site groupings, with additional pairwise post-hoc comparisons using BH corrections.

Additionally, we examined the time-resolved UniFrac distances between our selected samples to provide an additional spatial analysis which accounts for the temporal covariation within the data. For each time point, we examined the weighted and unweighted UniFrac distances between sample pairs (effluent to immediate downstream, effluent to upstream, effluent to further downstream, upstream to immediate downstream, upstream to further downstream, immediate downstream to further downstream). We then tested whether the combined distribution of time-resolved UniFrac distances between a given sample pair was significantly different between pre-intervention and post-intervention time points using a permutation test (999 permutations) [41, 45]. This test was repeated for all sample pairs, and the resultant *p*-values were adjusted using BH corrections. This entire process was repeated for sediment samples as well.

### Statistical analysis of fecal coliform data

Due to the skewed sampling distributions and the presence of substantial outliers in the measured fecal coliform data, we employed a non-parametric, median-based bootstrap hypothesis test [11, 29] to examine whether fecal coliform concentrations at sites downstream of WRPs significantly changed post-intervention. At each site, the median fecal concentration value from time points post-intervention were contrasted with the median fecal concentration value from time points pre-intervention to generate a test statistic. Next, the fecal coliform data were randomly resampled with replacement among all the time points to generate a simulated test statistic given the null hypothesis that fecal concentrations are non-associated with the implementation of the intervention in 2016. This process was repeated 10,000 times to generate a null distribution of test statistics, which could then be compared against the true test statistic in a two-tailed test for significance. This analysis was performed with effluent samples at both WRPs, water samples at sites immediately downstream from WRPs (Site 56 and 76), and water samples further downstream from the WRPs (Site 57 and 96).

### Statistical analysis of physicochemical data

We examined the effects of the wastewater disinfection treatments on the abiotic water characteristics of sites immediately downstream of the treatment plants. This was accomplished by testing for significant differences in key physicochemical variables (temperature, pH, Dissolved oxygen, NO_2_^−^/NO_3_^−^, NH_3_, PO4^3−^, volatile suspended solids, conductivity, and turbidity) between pre-intervention time points (2013–2015) and post-intervention time points (2016–2019). This analysis was conducted using site 76 (immediately downstream of Calumet), site 56 (immediately upstream of Calumet), site 36 (immediately downstream of O’Brien), and site 112 (immediately upstream of O’Brien) (Additional file 1: Fig. S5).

Like the fecal coliform data, most of the measured physicochemical variables contained skewed sampling distributions and substantial outliers. Moreover, the physicochemical variables were subject to seasonality, resulting in temporally autocorrelated data. Therefore, we employed another median-based bootstrapping simulation while also using the physicochemical measurements of upstream sites (Site 56 and 112) to account for seasonal variation. For each measured physicochemical variable of interest, differences in the measurements of upstream and downstream sites were calculated for each time point. Then, the median values of such measurements in post-intervention time points (2016–2019) were compared against the median values of such measurements in pre-intervention time points (2013–2016) to form a test statistic. These metrics were compared to a bootstrapped null distribution in which upstream and downstream measurements were drawn from the same sampling pool and the process was repeated to create simulated test statistics under null conditions. The true test statistic was compared against the null distribution in a two-tailed test for significance, and the total p-values were adjusted for multiple comparisons using Benjamini–Hochberg corrections.

## Results

### Microbial community compositions are well defined by sample type.

**Fig. 1:**
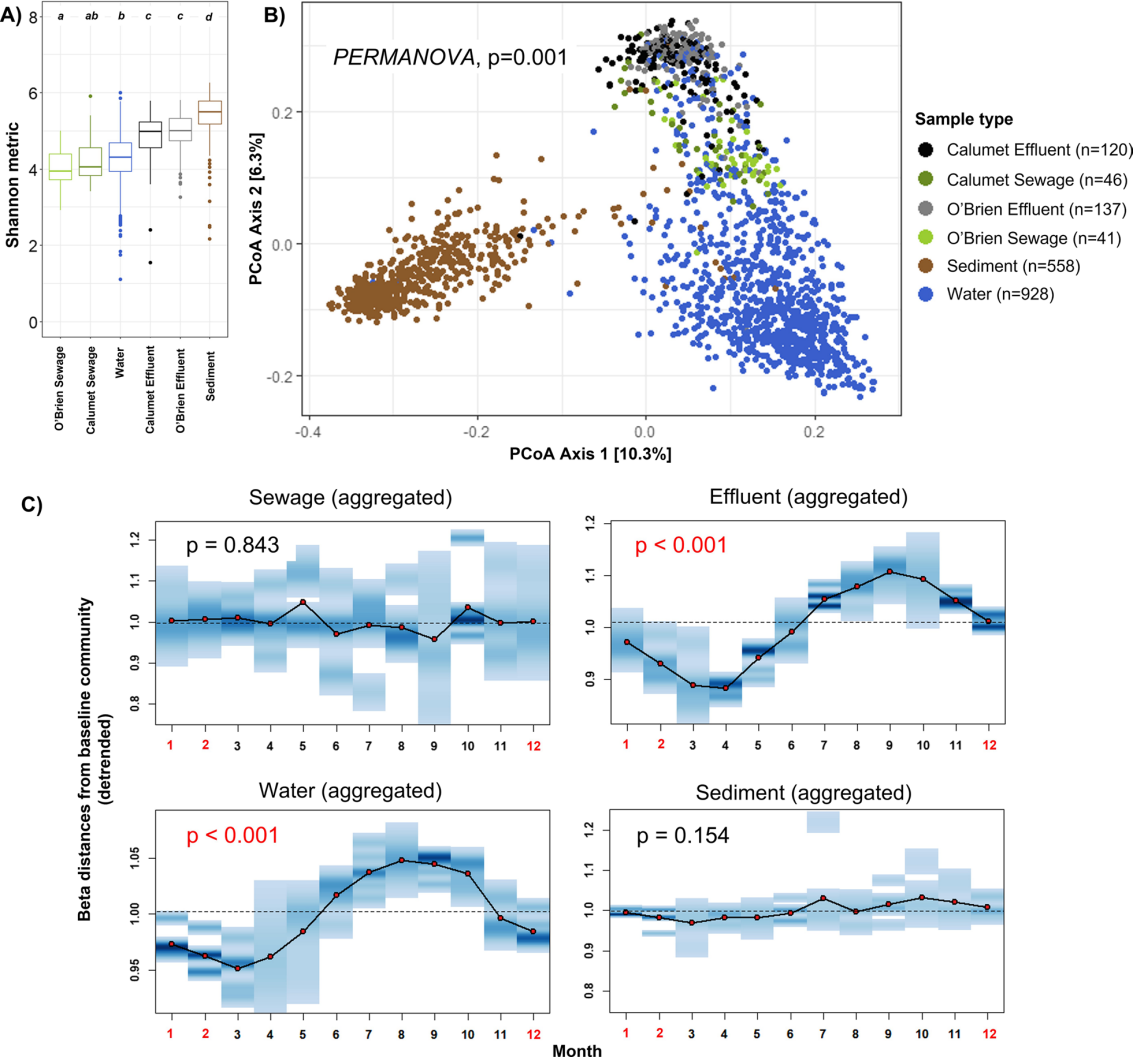
**A** Alpha diversity of CAWS samples by sample type. The distribution of Shannon diversity indices for each sample type is consolidated for all seven sampling years (2013–2019). This box and whisker plots demonstrate the quartile range and outliers for each distribution. Statistical groupings are designated by the letters above the boxplots. Significance was assessed using paired Wilcoxon tests with Benjamini–Hochberg multiple testing corrections. **B** Beta diversity analysis of CAWS samples by sample type. PCoA plot based on the unweighted UniFrac distance matrix showing clustering patterns of different sample types. **C** Aggregated seasonal plots of beta distances as a function of month, for each sample type. The dots connected by solid black lines represent the mean beta distance between each sample community and a fixed baseline community of the same sample type and site. The blue density bands describe the distribution of beta distances to a baseline community, for each particular month. The dashed line signifies the mean beta distance value of all samples relative to its baseline community for a given sample type, and represents the null expectations given that seasonality plays no role in the community. As no collections were conducted in December, January, and February (axis labeled in red), the values of these months were interpolated using spline regression

Microbial communities from each sampled medium (water, sediment, sewage, effluent) significantly differed in both alpha and beta diversity indices. Sample types displayed significantly different alpha diversity values from one another as measured by Shannon’s diversity index (Kruskal Wallis test, *p* < 0.001, Fig. 1A). As expected [14, 20, 43, 51, 58], sediment samples had the greatest alpha diversity, followed by effluent samples, then followed by water and sewage samples (pairwise Wilcoxon tests with BH corrections,See Fig. 1A for statistical groupings.) Notably, there were no significant differences in alpha diversity values between the effluent samples at O’Brien and Calumet, or between the sewage samples at O’Brien and Calumet, although alpha diversity differences between the sewage and effluent types were highly significant (*p* < 0.001).

Beta diversity analyses using both weighted and unweighted UniFrac distance metrics indicated significant differences in community composition across sample types (weighted UniFrac PERMANOVA *p* < 0.001, unweighted UniFrac PERMANOVA *p* < 0.001, Fig. 1B). This was supported further by pairwise post-hoc comparisons with Benjamini–Hochberg corrections, which showed significant differences in community composition between all pairwise combinations of sample types (Additional file 1: Table S2). Beta dispersion analyses across sample type (calculated as the mean value of unweighted UniFrac distances between all coordinates of individual samples to the coordinate of its respective sample type centroid) indicated that water samples contained the highest levels of spatio-temporal community variability (mean distance to centroid = 0.5759), followed by sediment (mean distance = 0.5499), Calumet sewage (mean distance = 0.5401), Calumet effluent (mean distance = 0.5309), O’Brien effluent (mean distance = 0.5198), and finally O’Brien sewage (mean distance = 0.5123) (Additional file 1: Fig. S2).

### Seasonal trends play a dominant role in shaping the CAWS effluent and river community, but not in the sewage and sediment community

The temporal dimension of microbial community dynamics varied across sites and sample media. Traditional ordination-based methods for community distance metrics (*e.g.,* PCoA) do not account for the inherent temporal autocorrelation within the data [32], therefore, we directly examined the UniFrac distances of each sampled community to a site-specific baseline community over time. We found that several of the site- and sample type-specific time series plots showed a yearly cyclical component (Unweighted Unifrac—Additional file 1: Fig. S2, Weighted UniFrac—Additional file 1: Fig. S3). When aggregating distance results across sites, we found evidence for seasonality in effluent and water but not sewage and sediment (Fig. 1C). This effect remained clear regardless of the use of unweighted UniFrac distance (effluent samples: Friedman test statistic = 61.00, *p*-value < 0.001; water samples: Friedman test statistic = 57.13, *p*-value < 0.001) or weighted UniFrac distance metrics (effluent samples: Friedman test = 57.13, *p*-value < 0.001; water samples: Friedman test = 47.62, *p*-value < 0.001). In comparison, we found no evidence for seasonality with sewage (unweighted UniFrac: Friedman test statistic = 6.44, *p*-value = 0.828; weighted UniFrac: Freidman test statistic = 10.64, *p*-value = 0.474) or sediment samples (unweighted UniFrac: Friedman test statistic = 15.67, *p*-value = 0.154; weighted UniFrac: Friedman test statistic = 13.38, *p*-value = 0.269).

**Fig. 2:**
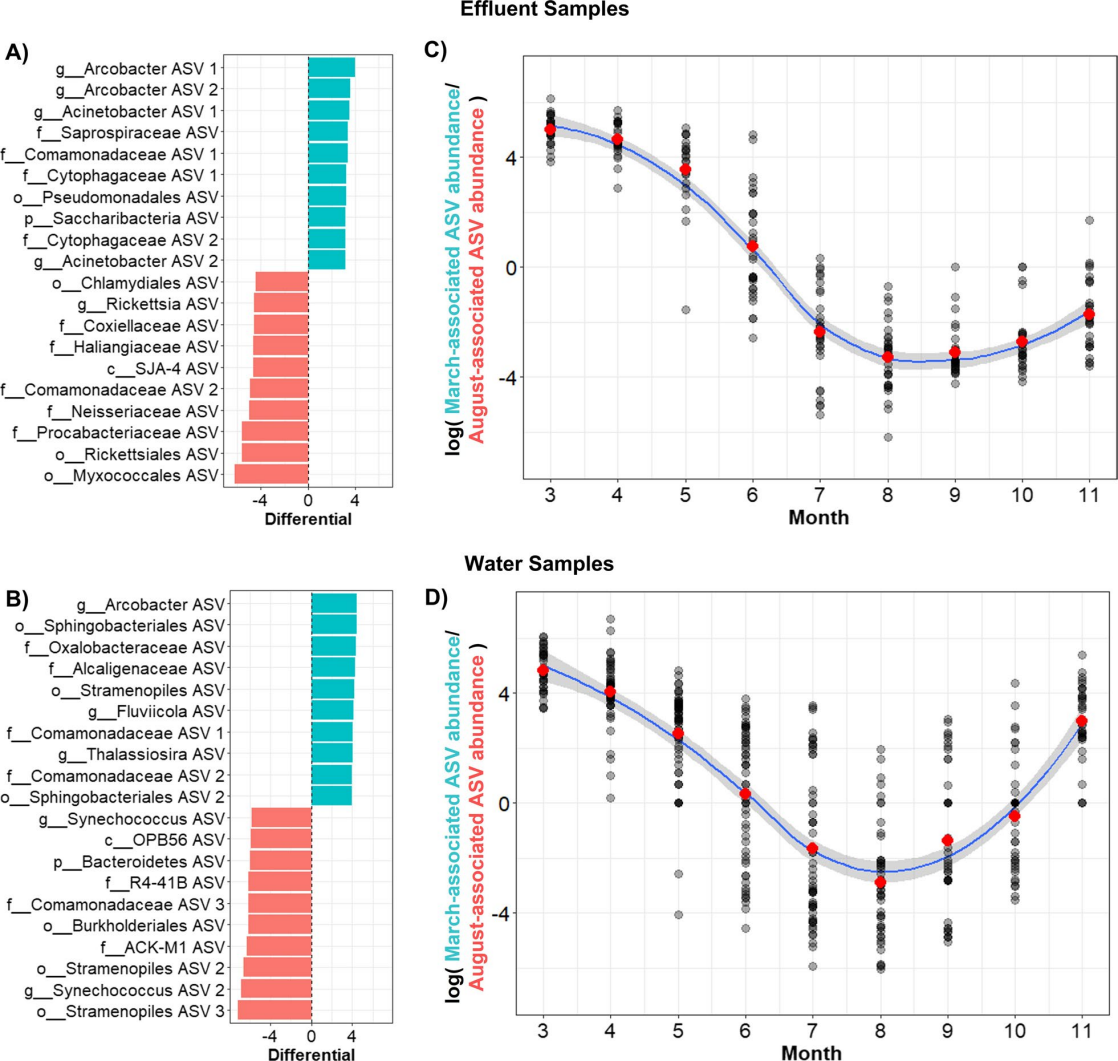
**A**, **B** Differential abundance plots of water and effluent samples, comparing samples collected in March to samples collected in August. Positive differential values indicate the top ten ASVs that were differentially abundant in March samples compared to August, and negative values indicate the top ten ASVs that were differentially abundant in August samples relative to March. **C**, **D** Log ratios of March-associated ASVs to August-associated ASVs as a function of month in water and effluent samples. Red points indicate the mean value of each month. Blue lines are best-fit curves of the data using a local polynomial regression fitting method (loess) with 95% confidence intervals

Water and effluent samples contained strong seasonal signals in community composition, therefore we performed compositionally-aware differential abundance analyses [38] with both sample types and characterized the specific taxonomic groups driving our observed changes in the community composition across seasons. Differential abundances in ASVs between March and August time points were calculated using Songbird models trained with subsetted data of only water and effluent samples collected at either month (effluent samples n = 63, water samples n = 197). The goodness-of-fit values of resultant models (effluent model pseudo-Q square = 0.161, water model pseudo-Q square = 0.103) indicated a strong effect size of month in contributing to model fit for both sample types (relative to null models). After identifying the top ten ASVs that increased most significantly in March relative to August (henceforth referred to as March-associated ASVs), and top ten ASVs that were differentially abundant in August relative to March (henceforth referred to as August-associated ASVs) (Fig. 2A), we examined the log-ratios of March-associated to August-associated ASVs across the entire time series (Fig. 2B). We found that such log-ratios indeed followed a gradual and predictable seasonal gradient through time when extrapolated onto the entire time series, providing validation that our identified ASVs follow strong seasonal shifts in relative abundance within their respective sample type.

### Spatially unique microbial communities experience a continuum of compositional shifts along the river

**Fig. 3:**
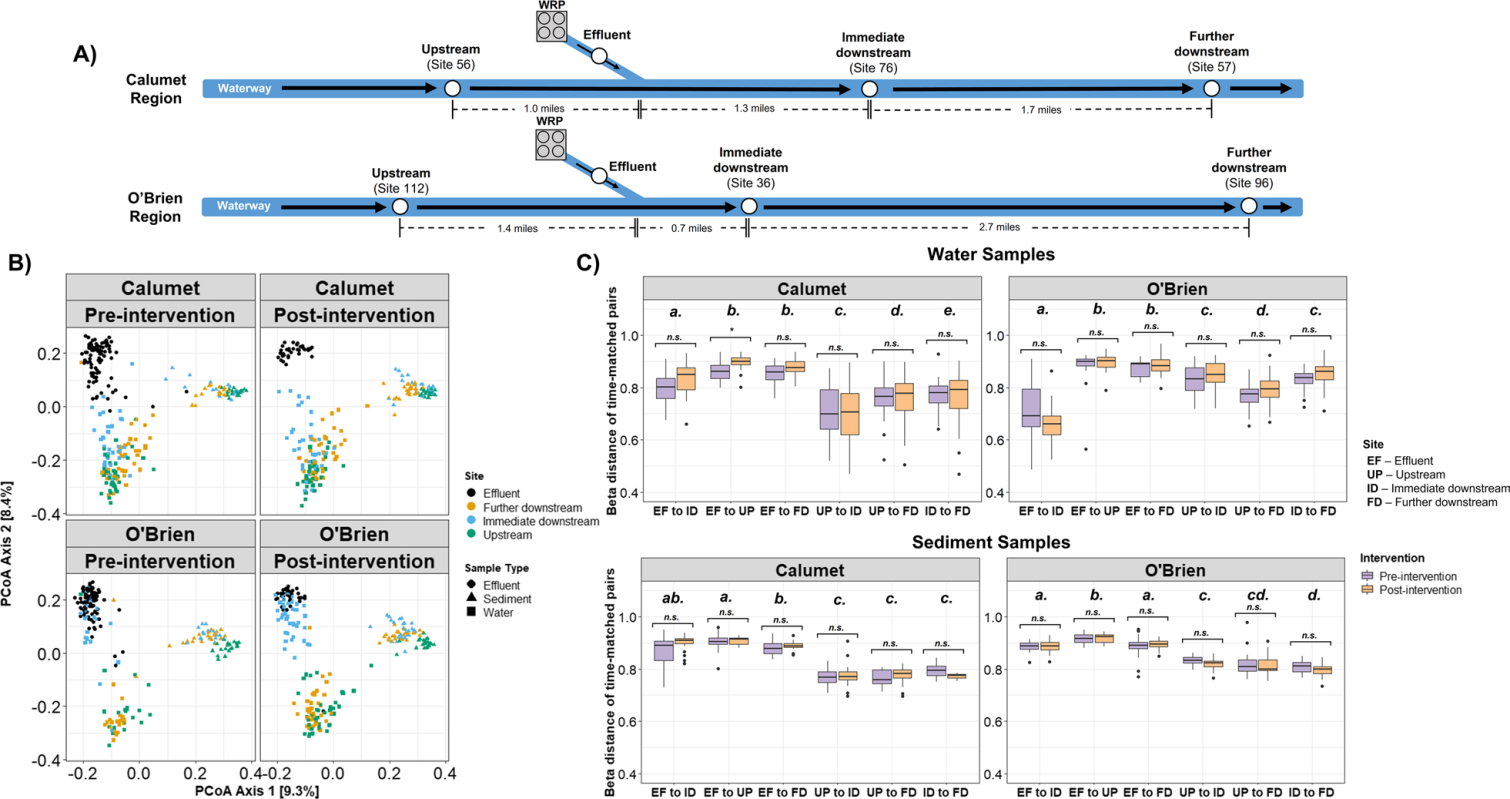
**A** Simplified map of the Calumet and O’Brien regions of the CAWS. Subsetted sites used in the following analyses are described in this map. **B** Unweighted UniFrac PCoA plots of effluent sample, and water and sediment samples from upstream, immediate downstream, and further downstream relative to WRPs. Plots are separated by region and by intervention period. **C** Unweighted UniFrac distances between pairs of sites, matched by time point. Statistical comparisons between pre- and post- intervention distances within each pair are denoted using brackets, with n.s. indicating non-significance (*p* > 0.05). Statistical groupings of comparisons across pairs are designated by the letters above the boxplots. Significance was assessed using t-tests with Benjamini–Hochberg multiple-testing corrections

Time-resolved spatial dynamics of the CAWS microbiome were characterized to examine the impacts of the WRP system upgrade interventions on downstream community dynamics. This targeted analysis was constrained to only include water and sediment samples of sites immediately upstream (1.0 miles away), immediately downstream (1.3 miles away), and further downstream (3.0 miles away) of Calumet WRP, sites immediately upstream (1.4 miles away), immediately downstream (0.7 miles away), and further downstream (3.4 miles away) of O’Brien WRP, and the effluent samples from both WRPs (Fig. 3A). Significant differences in microbial community structure were quantified between all pairs of sites for both water and sediment samples. (Fig. 3B, see Additional file 1: Table S3 for PERMANOVA results).

Examinations of time-resolved UniFrac distances between WRP-adjacent sites showed that community similarity measures were often consistent with the spatial orientation of site locations to one another and to the WRP outfalls, particularly with water samples (Fig. 3C). In the O’Brien region, water communities collected from the site immediately downstream of the WRP were significantly more similar to the effluent communities than to water samples upstream or further downstream. Further downstream from the O’Brien WRP, water communities became less similar to effluent samples and increased in resemblance to the upstream river community prior to WRP effluent outflow. We found similar trends in the Calumet region as well, with water communities immediately downstream of the WRP being the most compositionally similar to effluent compared to the other river sites. However, unlike the case in the O’Brien region, UniFrac distances of the immediate downstream site to effluent were significantly larger compared to its distances to the other river sites, likely owing to the fact that the immediate downstream site at the Calumet region is twice as far from the WRP outfall than at the O'Brien region.

Within sediment communities, we found that each site also contained compositionally distinct microbial communities whose differences remained robust through time (Fig. 3C). Additionally, we note that unlike in the water communities, sediment community similarities did not follow a spatial orientation consistent with site location; microbial communities from all sites had similar UniFrac distances to the effluent, for both the Calumet and O’Brien regions (Fig. 3C). Similar results were found when measuring distances with weighted UniFrac (Additional file 1: Fig. S5).

Despite the clear impact of wastewater effluent on immediate downstream water, we found minimal evidence that the WRP upgrades caused any significant changes in site-specific community compositions in either water or sediment. Comparisons of time-resolved beta distances between pre and post-intervention time points found a statistically significant change in distance for only one site pair: effluent to upstream river communities at the Calumet region (Fig. 3C). Re-analysis with weighted UniFrac distances also did not identify meaningful community changes as a result of the intervention (Additional file 1: Fig. S6). Unsurprisingly, differential abundance models attempting to identify key microbial ASVs that significantly changed in abundance between pre-intervention to post-intervention time points resulted in poor model fits (pseudo Q square scores < 0.05), indicating low effect sizes.

### The WRP upgrades are effective in reducing specific populations of coliform-forming bacteria in the river, yet observable effects quickly diminish downstream

**Fig. 4:**
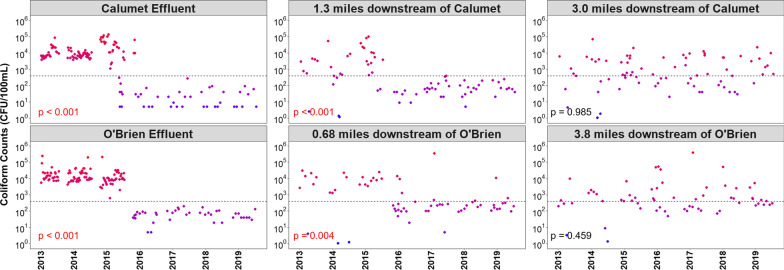
Log-transformed fecal coliform concentrations as a function of time in effluent and downstream river samples of Calumet and O’Brien WRPs. Plots on the left represent effluent samples directly from WRPs, plots in the center represent samples of river sites immediately downstream from WRPs, and plots on the right represent samples of river sites further downstream of the WRPs. The dotted line represents 400 CFUs per 100 mL, a concentration set as the EPA standard for recreational waters. The *p*-values represent results of bootstrapping simulations testing for differences in median values of coliform concentrations between pre-intervention time points (before 2016) and post-intervention time points (after 2016)

The spatio-temporal trends of fecal coliform counts cultured from water samples were analyzed. These counts are used as indicators of potential fecal contamination and as metrics for overall water quality for recreational and drinking use [34]. Throughout the study period fecal coliform concentrations (measured as the number of colony forming units per 100 mL of sample, or CFU/100 mL) were quantified from effluent and water samples. In the effluent samples, bootstrap hypothesis tests showed that median values of fecal coliform concentrations were significantly and substantially reduced following water treatment upgrades at both O’Brien (absolute effect size = 10,940 CFU/100 mL, *p* < 0.001) and Calumet (absolute effect size = 8280 CFU/100 mL, p < 0.001) sites. All pre-upgrade effluent samples contained fecal coliform concentrations that considerably exceeded the recreational water quality standard of 400 CFU/100 mL [55] while all post-upgrade effluent samples contained concentrations below this value. When examining coliform concentrations of river sites immediately downstream of Calumet and O’Brien WRPs, bootstrapping tests also found a significant reduction in coliform concentrations post upgrade,however, the effect size was weaker relative to the effluent samples and the signal contained higher noise (Calumet effect size = 1940 CFU/100 mL, *p* < 0.001, O’Brien effect size = 5690 CFU/100 mL, *p* = 0.004). Finally, examinations of coliform concentrations in sites further down the river contained no significant differences in concentrations between pre- and post- upgrade time points (*p* > 0.05), with levels fluctuating continually between below to above the EPA standard (Fig. 4).

### WRP upgrades did not significantly alter the physio-chemical environment of the downstream CAWS

Microbial community composition and dynamics are influenced by physicochemical parameters such as temperature, oxygen, nutrients, and pH. We tested whether WRP upgrades caused significant changes in key physicochemical water measurements in the downstream river sites by calculating the differences in physicochemical conditions at upstream and downstream sites relative to WRPs at each time point. We then tested for significant changes in such upstream–downstream differences between pre-intervention time points and post-intervention time points, using a median-based bootstrap hypothesis test (Table 1). Overall, we found no statistical support that the WRP upgrades caused significant shifts in physicochemical differences between upstream and downstream sites. In both the Calumet and O’Brien regions, we found no significant evidence that the intervention influenced upstream–downstream differences in any of the measured physicochemical variables. These comparisons of physicochemical parameters suggest the WRP upgrades played a relatively minimal role in affecting the physical and chemical conditions of downstream sites, particularly when compared to the natural seasonal variability captured at upstream sites (Additional file 1: Fig. S3).

**Table 1:** Results of median-based bootstrap hypothesis tests measuring for significant changes in physicochemical parameters between pre- and post- intervention time points, at Calumet and O’Brien WRP sites

	Calument region			O’Brien Region		
	Bootstrap test statistic	*p*-value	Adjusted *p*-value	Bootstrap test statistic	*p*-value	Adjusted *p*-value
Chlorophyll (ug/L)	1.6	0.433	1.000	− 0.6	0.807	1.000
Dissolved oxygen (mg/L)	0.25	0.514	1.000	0.215	0.623	1.000
NO_2_/NO_3_ (mg/L)	0.1745	0.486	1.000	0.52	0.585	1.000
NH_3_ (mg/L)	− 0.165	0.009	0.101	− 0.19	0.614	1.000
Total phosphorus (mg/L)	− 0.21	0.514	1.000	0.573	0.057	0.672
Volatile suspended solids (mg/L)	0	1.000	1.000	0	1.000	1.000
Total organic carbon (mg/L)	–0.2	0.284	1.000	0.05	0.762	1.000
Conductivity (ms)	0.0195	0.484	1.000	− 0.0355	0.560	1.000
Turbidity (NTU)	3.475	0.022	0.246	− 3.075	0.255	1.000
pH	0.145	0.051	0.557	− 0.075	0.479	1.000
Temperature (°C)	0.65	0.132	1.000	0.4	0.502	1.000

## Discussion

This study, which spanned 7 years from 2013 to 2019, aimed to characterize the spatio-temporal dynamics of microbial communities associated with the CAWS using 16S rRNA gene amplicon sequencing of samples collected from sediment, water, treated effluent, and raw sewage, along with fecal coliform counts and physicochemical measurements. Additionally, we investigated the impact of MWRD water quality improvement efforts (i.e. the WRP disinfection system upgrades and the implementation of the TARP system to capture CSOs) on the community dynamics of the CAWS microbiome.

As expected, compositional analysis of microbial communities demonstrated distinct distribution patterns across environmental media (river water, sediment, effluent, sewage), with significant differences between sample types for both alpha and beta diversity. As a noteworthy example, the microbial communities found in water samples contained relatively low alpha diversity values but the highest levels of spatio-temporal variability in community composition, consistent with expectations of high community turnover and shifting selection pressures typically found in aquatic environments [43, 48]. Sediment microbiomes were found to be most different from the other sample types (Fig. 1; as river water, sewage, and effluent all share the similarity of being aquatic environmental media, this reinforces the role of substrate type as a primary driver for community differences [28, 54].

Effluent samples and sewage samples significantly differed in both diversity-related and composition-related characteristics at both WRP sites, demonstrating the sizable impact of wastewater processing in affecting the microbial communities present in wastewater. This is consistent with findings that each step of the wastewater treatment process (primary, secondary, and final disinfection) often cause significant shifts in the resultant bacterial community that is eventually discharged into the river [23, 39]. Interestingly, we also note that samples from the two WRP sites were very similar to one another when examining both effluent and sewage sample types. Alpha diversity metrics were statistically indistinguishable between the two sites (Fig. 1) and pairwise PERMANOVAs found significant differences yet relatively low effect sizes when comparing site pairs of the same sample type (Additional file 1: Table S2); this indicates that both WRP regions receive similar microbial communities as sewage input, and that both treatment procedures tend to produce microbially similar effluent outputs despite using different disinfection strategies.

Spatiotemporal examinations of the CAWS community revealed that the wastewater treatment and CSO capture upgrades did not have dramatic effects on the overall community of the downstream river; rather, the effects were nuanced and population-specific. Proximity of water sampling location to effluent outfall exerted a clear influence on microbial community structure; for both Calumet and O’Brien, microbial communities in water immediately downstream of the outfall were consistently and significantly more similar to effluent than communities from other water samples. This effect was most pronounced for O’Brien, where the “immediate downstream” site was only 0.7 miles downstream of the outfall, compared to the closest downstream site to the Calumet outfall which was 1.3 miles. Indeed, for the O’Brien system, the immediate downstream water samples contained microbial communities that resembled effluent more than they resembled upstream or downstream water from the river, while the site immediately downstream of the Calumet outfall was influenced by effluent to a lesser degree and most closely resembled the site upstream. At sites further downstream, communities were found to display decreased community resemblances to effluent and instead began to return in similarity to the original upstream communities. Similar results were found by Pascual-Benito et al. [42], suggesting that freshwater bacterial communities may be able to re-establish relatively quickly following the impact of an effluent discharge. We also note that despite the wastewater treatment system upgrades at both WRPs in 2016, UniFrac distances between downstream water and effluent microbial communities remained consistent before and after the upgrades, suggesting that these upgrades did not exert a strong influence on changing the relative compositions of downstream microbial communities.

Measurements of fecal coliform concentration, a traditional standard of freshwater quality [18, 36, 55], demonstrated the clear success of the intervention in reducing the absolute abundance of fecal-associated bacterial taxa from effluent samples. This was also found to be the case with downstream river sites,yet, similarly to the beta distance analysis, effectiveness tended to diminish with respect to the samples’ distance from the WRP sites. In fact, sites greater than 3 miles away from either WRP no longer had any detectable differences in fecal coliform concentrations between pre- and post-intervention time points, remaining at a level comparable to pre-intervention timepoints and frequently exceeding the EPA recreational water threshold. Such results may indicate the presence of other sources of fecal coliform-associated bacteria to the waterway besides the WRP effluent and pre-TARP CSO events. We additionally note that this described spatio-temporal pattern was nearly identical between the Calumet and O’Brien regions, suggesting that the impacts of disinfection strategy (chlorination or UV disinfection) at these sites tended to become equally negligible once discharge traveled several miles downstream.

In contrast to fecal coliform results, we found that the overall dynamics of CAWS microbial communities appeared robust and consistent over the course of the study. Neither PERMANOVA nor time-resolved beta distance analyses using site- and sample-specific comparisons revealed any significant impact of the system upgrades in the compositional community dynamics of downstream river sites. Instead, the predominant driver of microbial community composition was a consistent yearly cyclical trend, a trend which we attribute to seasonal effects driving the ecology of the CAWS microbiome. This was particularly the case with effluent and water samples, while sediment and sewage communities remained consistent throughout each year. Evidence for seasonal effects have been observed in various aquatic-based microbiomes, most prominently in marine systems [5, 16] but additionally in freshwater river systems [52, 62] and in the activated sludge of wastewater systems [21, 22, 44]. In our study we found that such seasonal effects overpowered any potential effect of the intervention in shifting community dynamics of downstream river sites. Similar examples have been noted in aquaculture systems [30, 61], indicating the pervasiveness of seasonality as a major contributor to microbial community structure. We also found that there was relatively minimal overlap in the specific ASVs most affected by seasonality when comparing between effluent and water communities, suggesting that microbial populations can be differentially affected by seasonal trends based on community- and environment-specific factors.

Notably, we found that sewage and sediment did not appear to be affected by seasonal trends in our study (although there were a few exceptions of site-specific sediment communities that tested significantly for seasonality). Of particular note is our finding that the sewage communities in both O’Brien and Calumet WRPs did not appear to contain a discernible relationship to time of year, despite the highly consistent seasonal signal found in the effluent. As the microbial communities within activated sludge of engineered bioreactors have demonstrated seasonal qualities [21, 22, 44], we speculate that exposure to the wastewater treatment process in our system may introduce a seasonal component to the microbial communities as they transition from untreated sewage to treated effluent, although confirmation would require a more nuanced study. We also note that such results contrast with other work examining the sewage microbiome through time in Milwaukee, WI [25], which found a seasonal signal that was well correlated with variations in wastewater temperature. We expect that differences in various factors such as treatment plant size, sewage volume, sewer interceptor design, and particularly sewer line depth may have reduced our sewage samples’ exposure to seasonality-inducing factors such as temperature variability, allowing for an increased exposure to factors that are more randomly distributed through time. As for the sediment, communities were found to be relatively stable through time and instead were most definable by site, suggesting that despite their close proximity to water, their dynamics are distinct from those of water.

## Conclusions

River systems are unique environments for examining microbial community dynamics, in that they offer spatial complexity that is integrated with temporal patterns. In this seven-year study, we characterized the microbial community of the CAWS across a broad spatio-temporal gradient and described its response to a prominent environmental change. Our results from this seven-year long microbiome study are nuanced; on one hand, we provide evidence that wastewater management improvement efforts such as implementation of large scale disinfection technologies and combined sewer overflow capture systems can lead to significant improvement in water quality, as indexed by reductions in population sizes of known indicators of fecal contamination. On the other hand, our analyses indicate that such water quality improvement measures do not appear to greatly shift the structure of existing microbial community dynamics in the waterway overall, or the physicochemical environment in which the microbial communities exist. Our results demonstrate the robustness of community dynamics in the system despite an interventional disturbance that significantly reduced the prevalence of fecal coliform-associated bacteria immediately downstream of the WRPs.

## Supplementary Information

**Additional file 1**.** Figure S1**. Chicago Area Waterways System (CAWS) and Water Reclamation Plant (WRP) sampling locations.

## Data Availability

The datasets generated and/or analysed during the current study are available in the European Bioinformatics Institute repository, [ERP136279]. Additionally, sequencing data and processed tables and taxonomy assignments are available through QIITA under study ID14446.

## Glossary

CAWS: The Chicago Area Waterway System.
WRP: Wastewater reclamation plant. There are two WRPs in the system, named O’Brien and Calumet.
Sewage: Incoming untreated wastewater entering WRPs.
Effluent: Outgoing treated discharge leaving WRPs.
CSO: Combined Sewer Overflow event, leading to discharges of untreated sewage directly into the CAWS.
TARP: Tunnel and Reservoir Plan, aimed to reduce the number of CSO events.
Intervention: The implementation of disinfection upgrades at WRPs as well as TARP upgrades to the CAWS system in 2016.
