# Supplementary material for: Microbiome response in an urban river system is dominated by seasonality over wastewater treatment upgrades
Source: Environ Microbiome. 2023 Feb 19;18:10. doi: 10.1186/s40793-023-00470-4 (PMC9938989; doi:10.1186/s40793-023-00470-4)
Supplement: Supplementary file 1 — Additional file 1. Figure S1. Chicago Area Waterways System (CAWS) and Water Reclamation Plant (WRP) sampling locations. [file 40793_2023_470_MOESM1_ESM.docx]

**Supplementary Information**


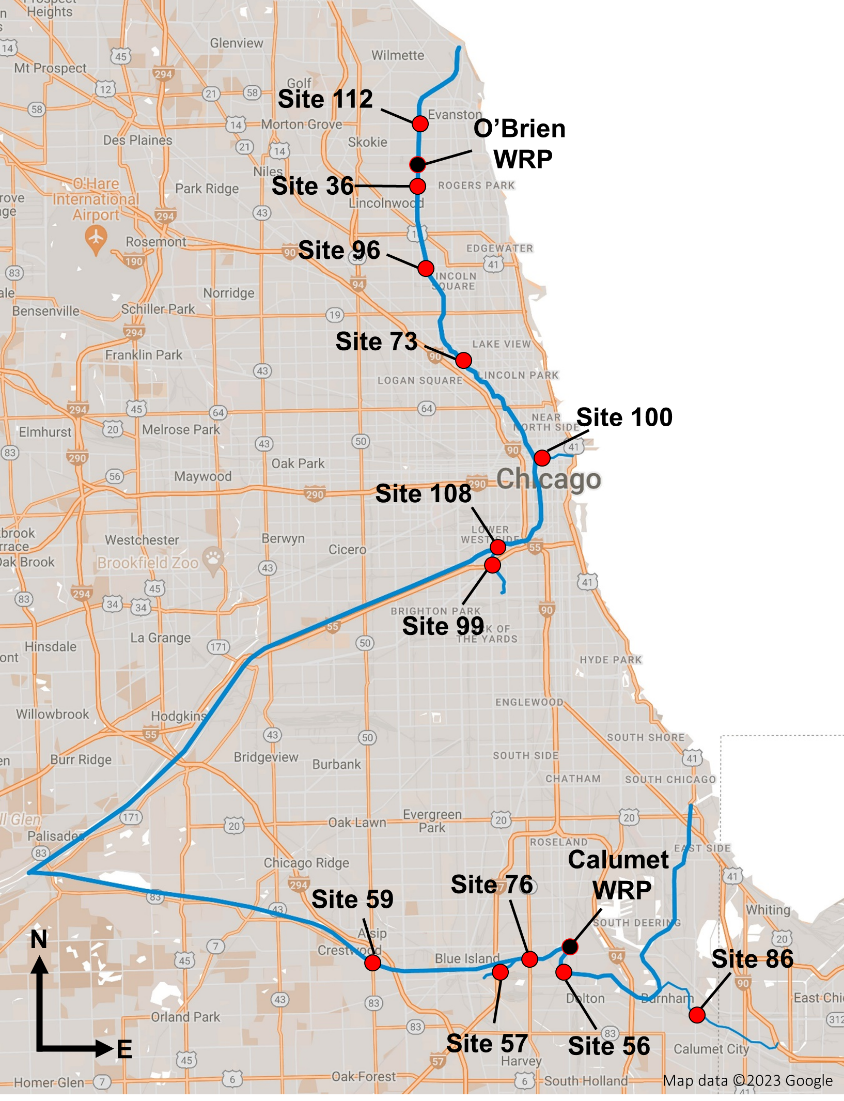


**Figure S1: (A)** Chicago Area Waterways System (CAWS) and Water Reclamation Plant (*) sampling locations. Tributaries feeding to the CAWS are in bold and upstream sites are in italic fonts.


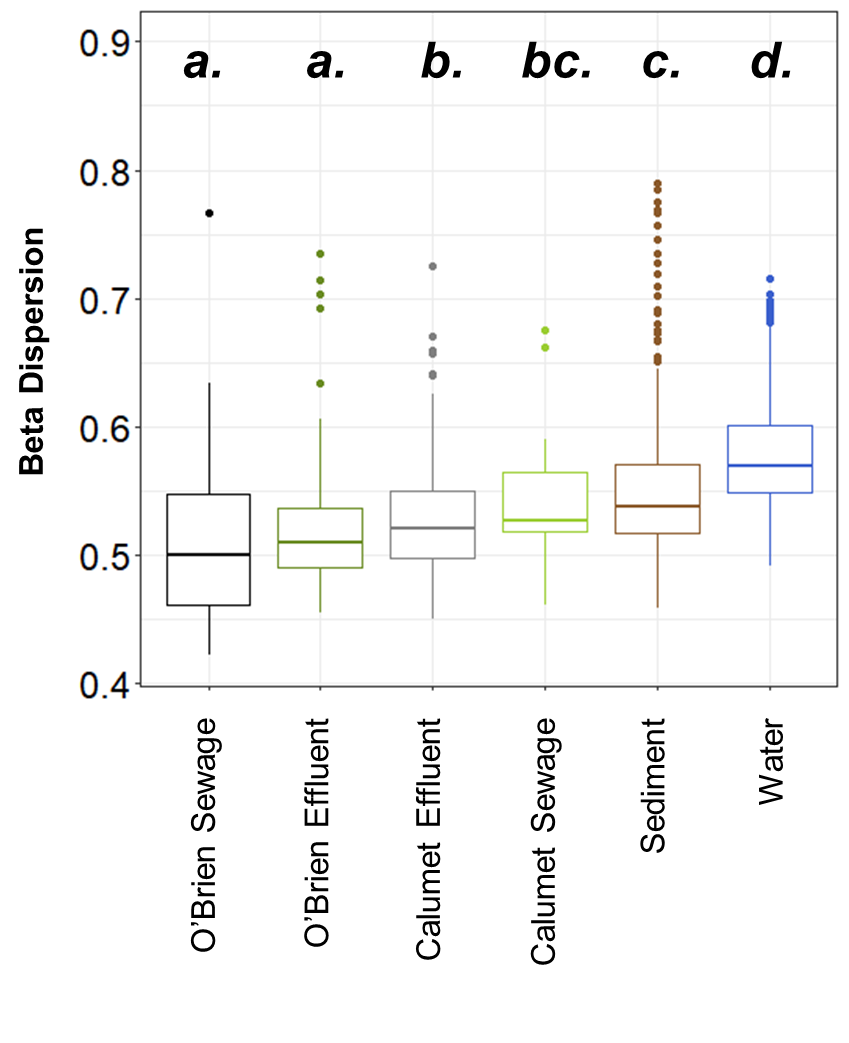


**Figure S2: Beta dispersion values of CAWS samples by sample type.** Beta distances were calculated using unweighted UniFrac distances.

***
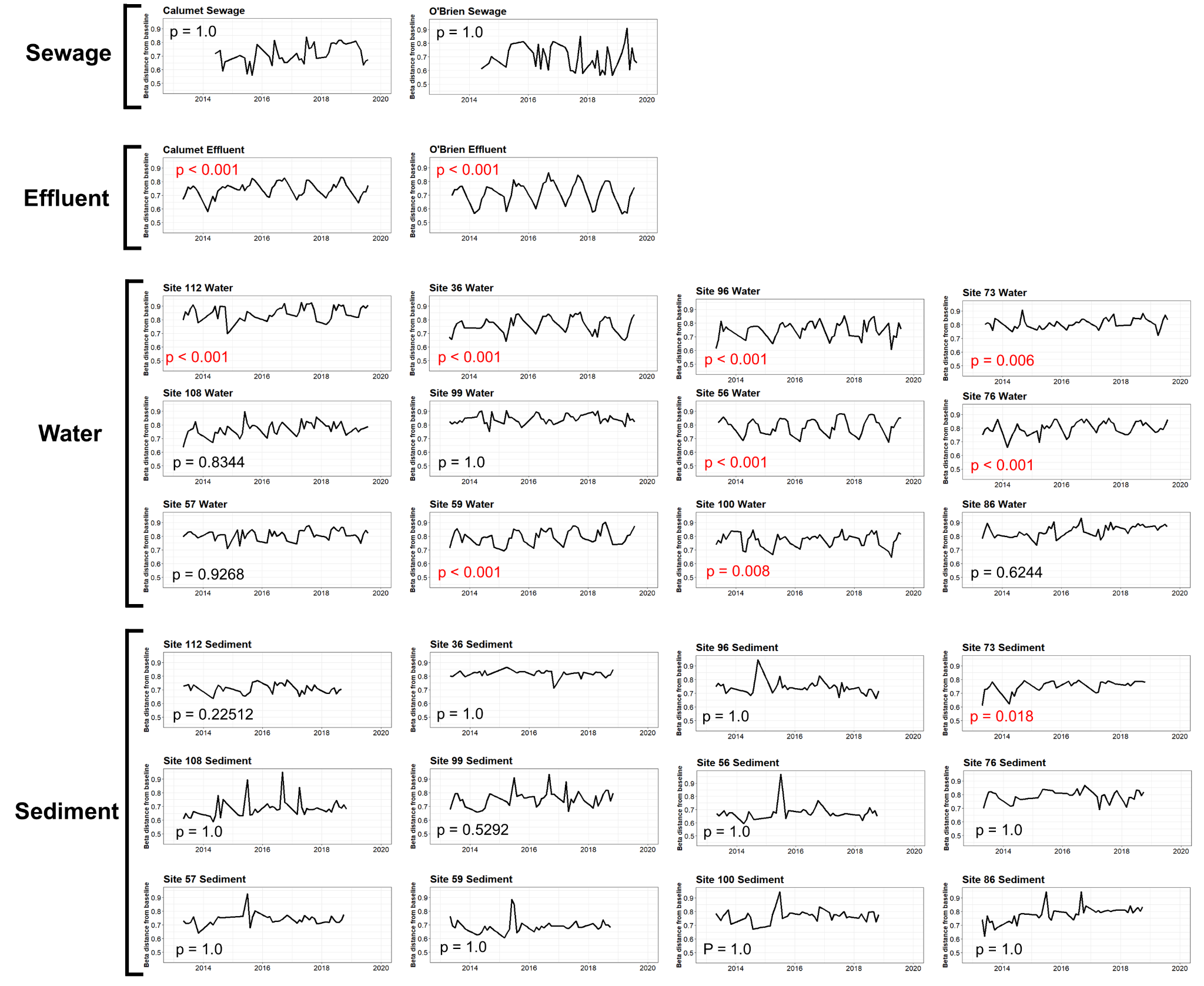
***

**Figure S3: Time series plots of Unweighted UniFrac distances from a baseline community over time, for each sample type and region.** Baseline communities were set in March 2014 for effluent, water, and sediment samples, and set in March 2015 for sewage samples. Then, the unweighted UniFrac distances of all subsequently collected samples to its original baseline community were calculated and plotted over time. Missing time points were interpolated using spline regression.

***
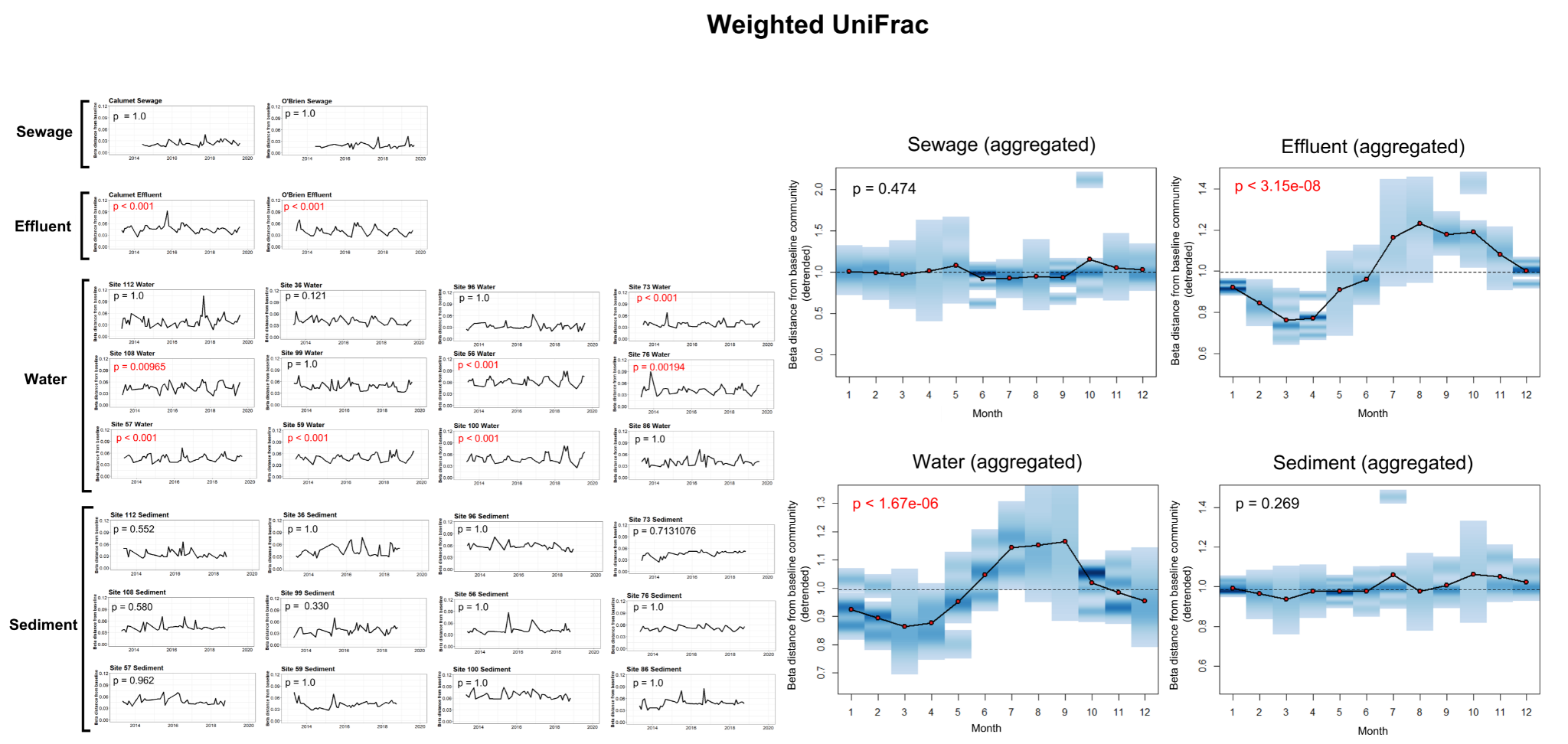
***

**Figure S4:** Weighted UniFrac distance time series plots.

**
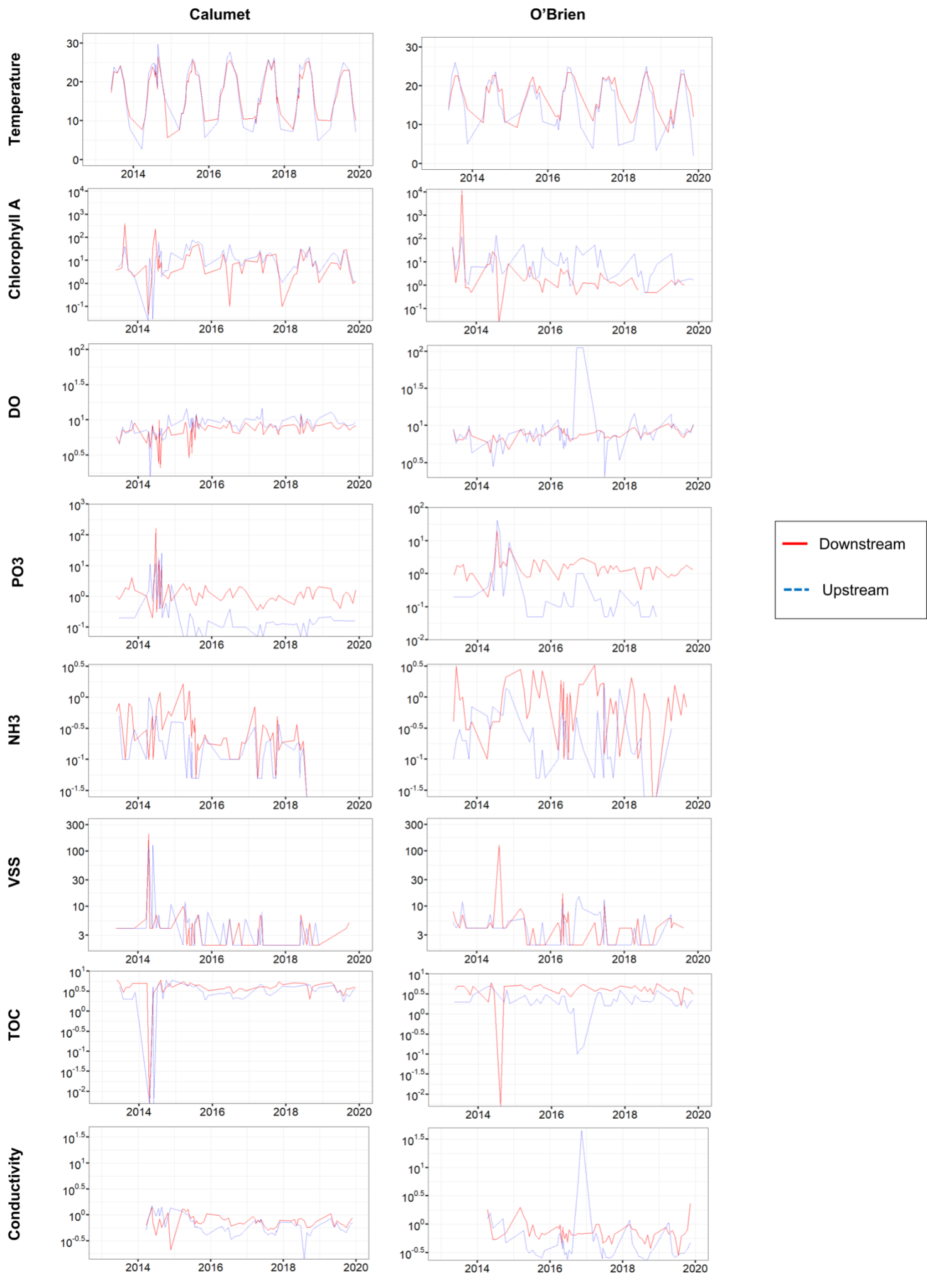
**

**Figure S5:** Plots of environmental variables vs. time at both upstream (red) and downstream (blue) sites for all measured physicochemical variables. Plots on the left represent sites in the Calumet region, and plots on the right represent sites in the O’Brien region.


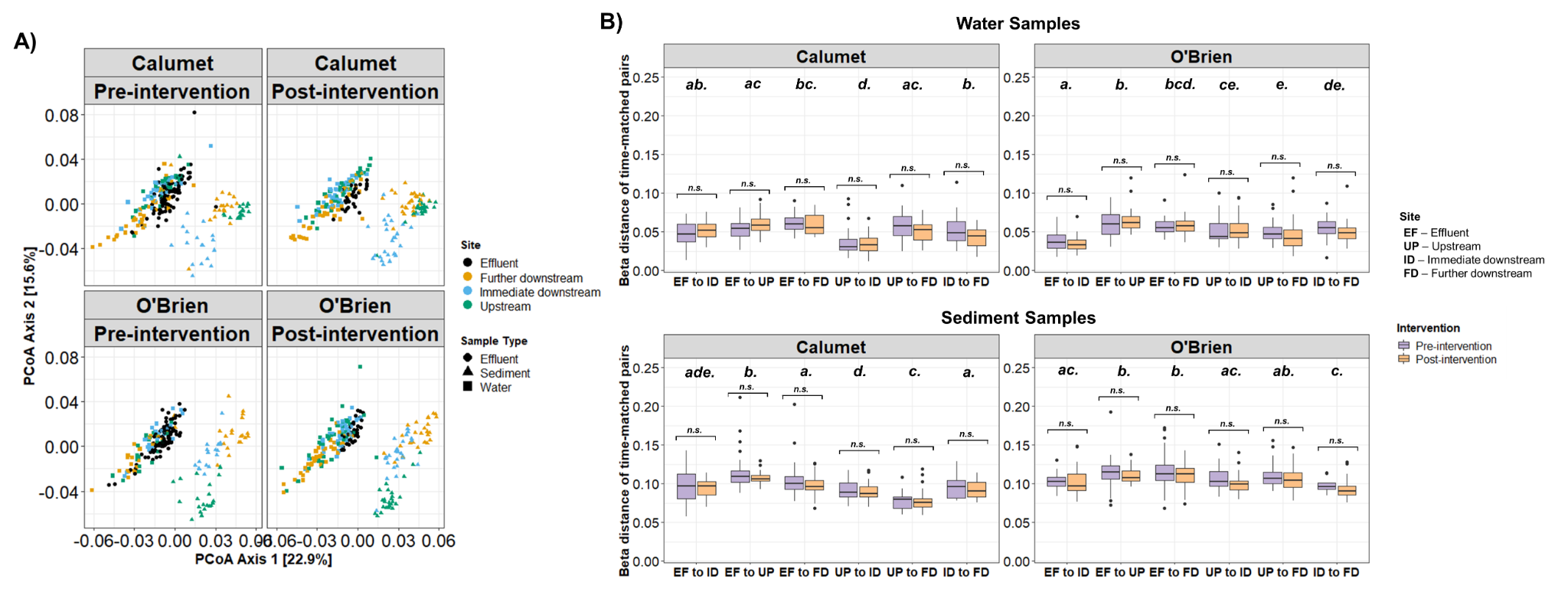


**Figure S6:** (B) Weighted UniFrac PCoA plots of effluent sample, and water and sediment samples from upstream, immediate downstream, and further downstream relative to WRPs. Plots are separated by region and by intervention period. (C) Weighted UniFrac distances between pairs of sites, with samples of matching time points. Statistical comparisons between pre- and post- intervention distances within each pair is denoted using brackets, with n.s. indicating non-significance (p>0.05). Statistical groupings of comparisons across pairs are designated by the letters above the boxplots. Significance was assessed using t-tests with Benjamini-Hochberg corrections.


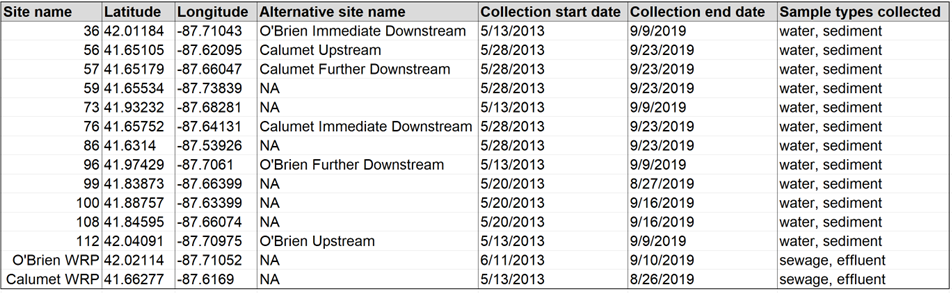


**Table S1:** Sampling campaign details of the study.


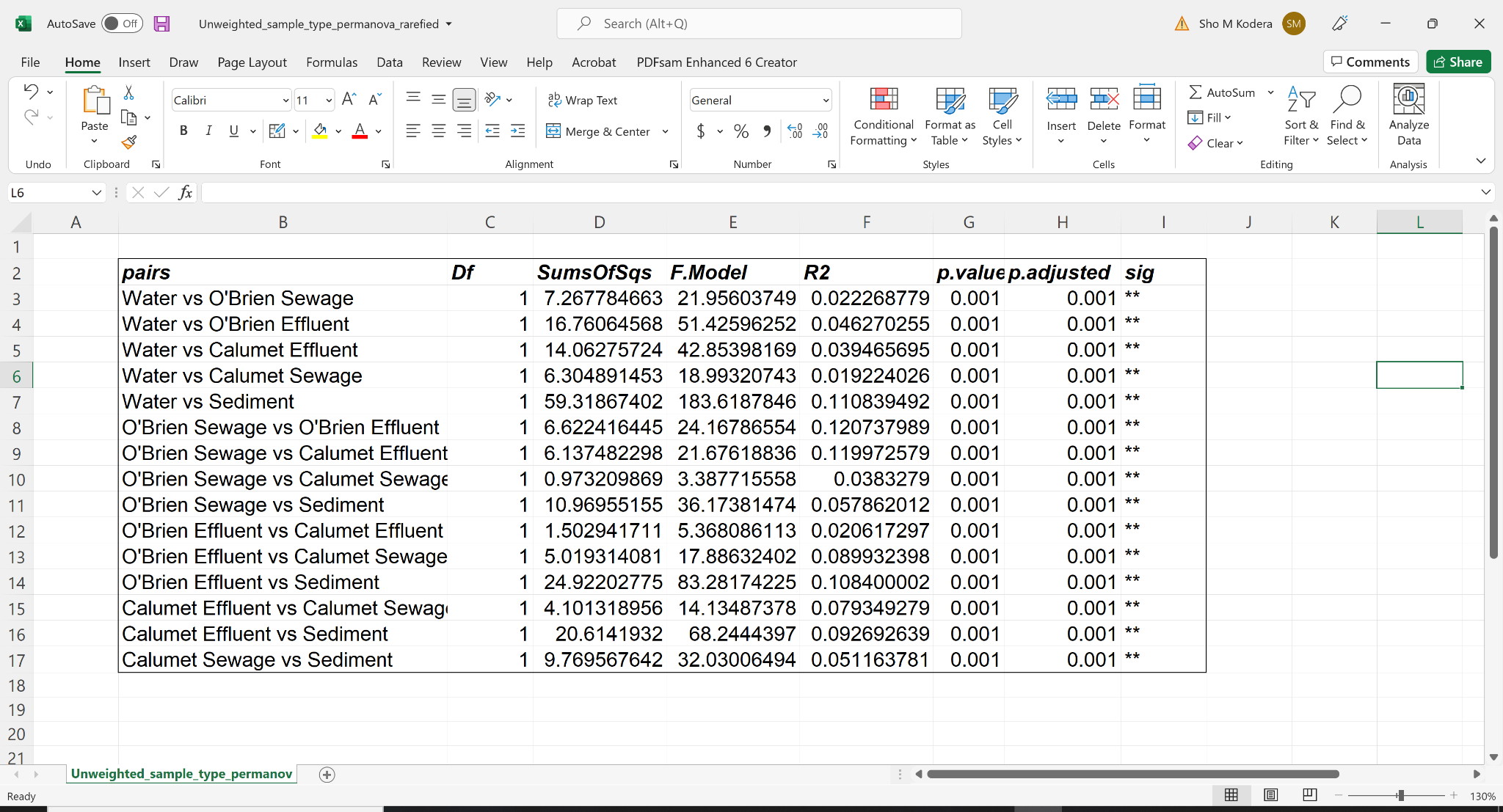


**Table S2:** Pairwise PERMANOVA results of tests for significant differences in community compositions between sample types, as depicted in Figure 1. P-values were adjusted using Benjamini-Hochberg corrections.


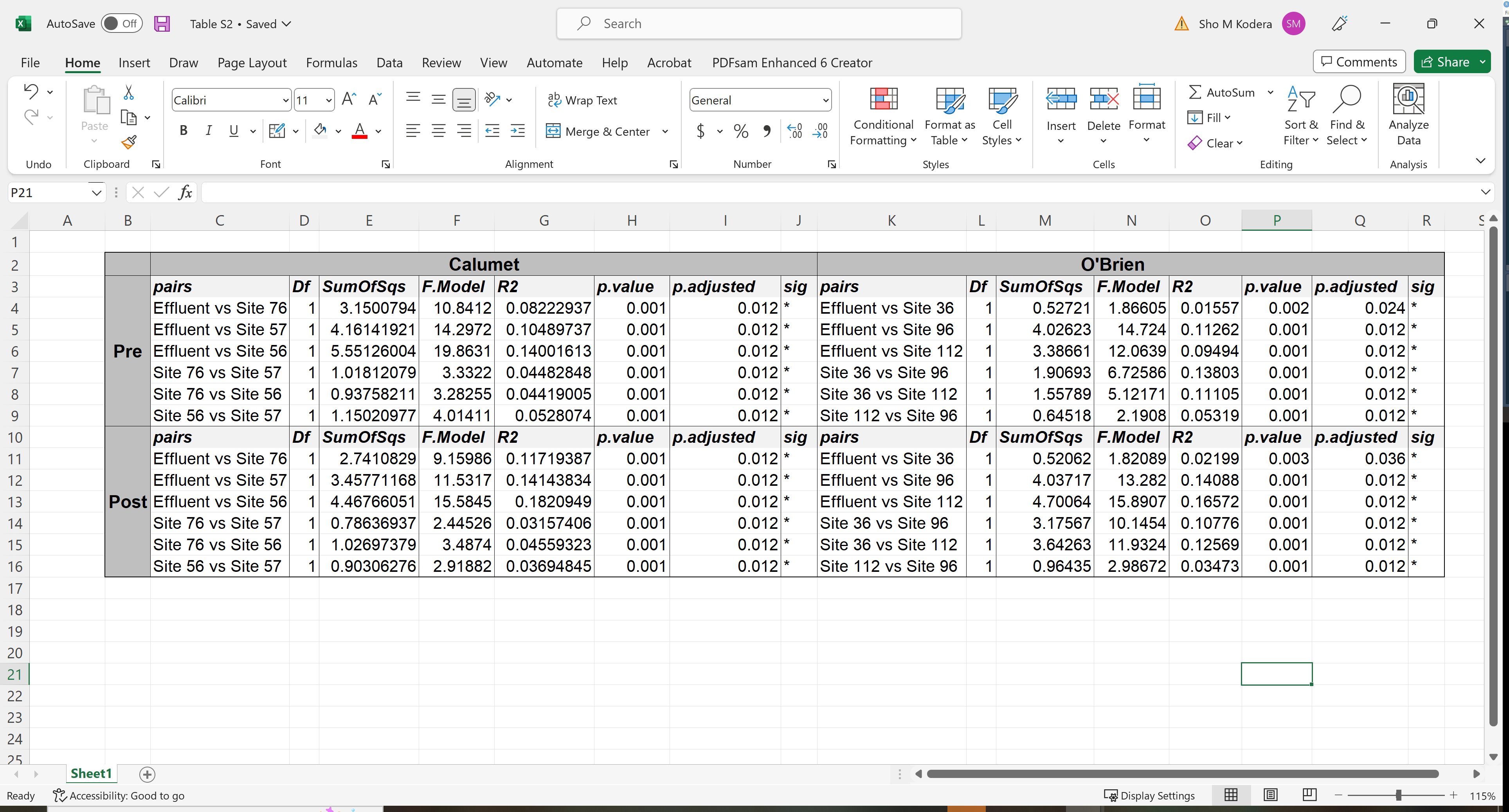


**Table S3:** Pairwise PERMANOVA results of tests for significant differences in community compositions between selected sites from Figure 3, nested by pre- and intervention time points. P-values were adjusted using Benjamini-Hochberg corrections.
